# Videos of demonstration versus text and image-based material for pre-skill conceptualisation in flipped newborn resuscitation training for medical students: a pilot study

**DOI:** 10.1186/s12909-022-03926-2

**Published:** 2022-12-05

**Authors:** Farah Yoosoof, Indika Liyanage, Ranjith de Silva, Savindra Samaraweera

**Affiliations:** 1 Department of Paediatrics, General Sir John Kotelawala Defence University, Rathmalana, 10390 Sri Lanka; 2 Sri Lanka College of Obstetricians and Gynaecologists, No.112 Model Farm Rd, Colombo, Sri Lanka; 3 Leeds General Infirmary, Leeds, UK

**Keywords:** Flipped classroom, Pre-class preparation, Pre-skill conceptualization, Newborn resuscitation, Medical students, Demonstration videos, Procedural knowledge

## Abstract

**Background:**

The flipped skills lab is a student-centred approach which incorporates pre-class preparation (pre-skill conceptualization) followed by repeated, hands-on practice for practical skills training. Objective measures of skills acquisition in the flipped literature are few and conflicting. The importance of pre-skill conceptualization in flipped skills training suggests that pedagogically informed pre-skill conceptualization can enhance outcomes.

**Methods:**

A mixed quasi-experimental study was conducted on 41 final year medical students who followed a flipped newborn resuscitation skills lab. Pre-class preparatory material covered conceptual and procedural knowledge. Students in the traditional group (n = 19) and those in the interventionalmental group (n = 22) received identical reading material covering conceptual knowledge. Procedural knowledge was shared with the interventional group as demonstration videos, while the traditional group received a PowerPoint presentation with text and images covering the same material. Knowledge acquisition was assessed by 20 single best answer questions before and after hands-on practice in the skills lab and skill performance was tested post-intervention with a simulated scenario. Students’ perceptions were collected by survey. Quantitative data was analysed using Wilcoxon Signed Ranks test and Mann–Whitney U test as appropriate. Qualitative data was analysed by thematic analysis.

**Results:**

Overall student rating of the intervention was positive with ratings of 4.54 and 4.46 out of 5 by the traditional group and the experimental group respectively. Post-intervention skill performance in the experimental group was significantly better (*p* < .05) in the interventional group (M = 87.86%, SD = 5.89) than in the traditional group (M = 83.44, SD = 5.30) with a medium effect size (r = .40). While both groups showed significant knowledge gains, only students in the experimental group showed a statistically significant gain in procedural knowledge (*p* < .05) following the flipped skills lab. Finally, while both groups self-reported feeling more knowledgeable and confident following the intervention, the level of confidence was superior in the experimental group.

**Conclusions:**

Flipping the skills lab with pre-skill conceptualisation combining text-based conceptual knowledge and video-based procedural knowledge followed by simulation-based hands-on practice improves procedural knowledge and skills acquisition in newborn resuscitation training for medical students. This study shows that in addition to temporal benefits, pedagogically informed pre-skill conceptualization can confer procedure-specific cognitive and emotional benefits supporting skills acquisition.

**Supplementary Information:**

The online version contains supplementary material available at 10.1186/s12909-022-03926-2.

## Background

The Flipped Classroom (FC), also known as the inverted classroom, is a learner-centred pedagogical approach in which the usual order of educational activities is reversed. Traditionally, didactic knowledge sharing occurs during the class, followed by the assignment of knowledge application activities as homework. In contrast, a flipped approach allows specified educational material to be shared before the class for independent, self-regulated pre-class preparation by the student. In this way, in-class time is preserved for instructor-guided, active learning opportunities such as knowledge application and hands-on practice within a collaborative environment [1].

While systematic reviews have shown that the FC results in knowledge gains comparable or superior to other methods [2–4], evidence with regard to skills acquisition appears to be conflicting [2, 5, 6]. Despite this, promising results of recent flipped approaches to simulation-based procedural skills (PS) training [7–11] support the use of the FC framework in simulation-based education [12, 13]. However, behavioural frameworks used in simulation-based PS training, flipped or otherwise, which mandate repeated mechanical practice in a simulated setting may not be adequate for teaching complex PS [14]. Incorporation of elements of experiential learning as in deliberate practice with mastery learning have resulted in superior skills acquisition, retention and transfer to clinical practice [15]. While empirical evidence has demonstrated the effectiveness of such frameworks for simulation-based newborn resuscitation (NR) [16], the benefits are not without considerable investment in terms of teaching time, effort and resources [17].

Literature also suggests that besides repeated practice, skills acquisition and professional performance requires adequate background conceptual knowledge (CK) (theory and facts), procedural knowledge (PK) (how and why) and conditional knowledge (when and why to apply CK and PK) related to the skill [18, 19]. However, didactic sharing of knowledge during the skills practice session reduces the time available for hands-on practice. Additionally, when shared simultaneously with practice it can overload the learner by dividing the learner’s attention between the task of absorbing new information and hands-on practice [20], potentially leading to learning inhibition. A flipped skills lab (FSL) incorporates the flipped approach for PS training by sharing related CK and PK with the students prior to the hands-on practice session in the skills lab. This not only ensures that time in the skills lab is reserved exclusively for undisturbed hands-on practice but also helps in the efficient management of cognitive load [14]. Such provision of related knowledge in the form of underlying theory, norms, algorithms etc. in PS training is termed pre-skill conceptualisation [14].

However, NR is a complex PS necessitating considerable preparation with difficult material, posing a challenge for self-study. Given that the success of the FC is conditional on student preparation [21], extensive and difficult pre-skill conceptualisation can be detrimental to its success. Fortunately, carefully designed content can optimize preparation by reducing cognitive load and positively influencing student engagement and motivation [22]. While manuals [23, 24], PowerPoint presentations (PPs) [25, 26] and video lectures [27, 28] have been used to present various types of knowledge in flipped PS training, instructional theory suggests that PK is better learnt by the observation of a demonstration of the skill being performed [29]. Although demonstration videos (DVs) have been used as pre-skill conceptualisation in simulation-based flipped PS training in a handful of studies [7, 8, 30–33], singly or in combination with other material, only 3 of these reported on objective measures of skills acquisition and presented conflicting results [7, 8, 31]. Thus, there is a dearth of empirical evidence on the efficiency of pre-skill conceptualisation with DVs sharing PK for skills acquisition in the flipped approach to simulation-based PS training.

### The flipped newborn resuscitation training program

**Fig. 1:**
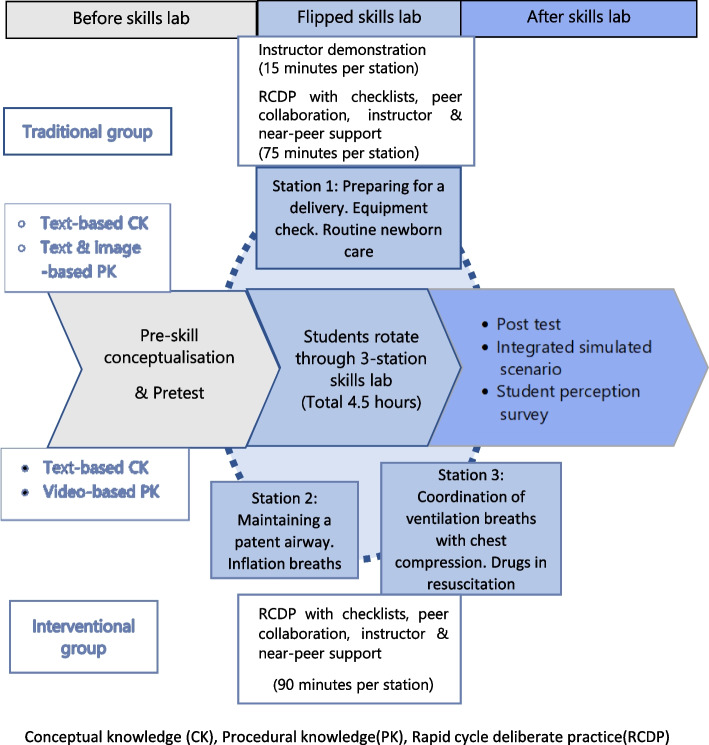
Comparison of the traditional newborn resuscitation skills lab with the flipped skills lab

Competency in advanced NR is one of the learning outcomes of the 8-week Paediatrics rotation for final year medical students as per the core curriculum in Paediatrics for Sri Lankan Medical schools [34]. Owing to the vast content that needs to be covered during this rotation, NR training is usually conducted as a single full-day session with a limited number of instructors. Taking into account the practical and pedagogical benefits of the flipped approach, a NR FSL was developed to cover the expected critical competencies related to newborn care and resuscitation. Figure 1 compares the traditional skills session with the NR FSL.

## Aim

The primary aim of this study was to compare DVs with PPs to share PK as pre-skill conceptualisation in flipped NR training for medical students. Based on the arguments presented above, we hypothesised that PK presented in the form of segmented, short DVs with step-by-step narration are superior to text-based PP presentations for the acquisition of NR skills. The secondary aim was to gather student views and feedback on the newly developed program.

## Methods

### Sample and setting

A convenience sample of consenting students was drawn from two consecutive student groups following the final year Paediatrics rotation in February 2022 and March 2022 at University Hospital General Sir John Kotalawela Defence University (UHKDU) where the NR FSL was conducted. None of the students had any previous exposure to NR or the FC.

### Design

A mixed quasi-experimental study design was used. The students received a brief explanation of the FC approach prior to the intervention by FY and IL. They were also informed about how the NR FSL and the study would be conducted and that the purpose of the study was to evaluate the NR FSL for quality improvement. Students were assured that non-participation in the study would not affect their academic standing. Students who attended the NR skills session in February 2022 were allocated to the traditional group (TG) while those who attended in March 2022 were allocated to the interventional group (IG). Data was collected online using Google forms with students identifying themselves by student registration number only, thus blinding the authors to the identity of the consented students. All students were required to submit the completed pre-test 6 h prior to the scheduled skills lab as a mandatory prerequisite for participation. The skills lab and post-intervention skills assessment were also mandatory for all students as part of the curriculum. Informed consent for the study was taken as a positive response to the question “Do you wish to participate in this study?” which was the first question in the Google form for the post-test. Students were assured that only complete data sets associated with the registration numbers of students who gave their consent in this way would be analysed. Submission of the completed student survey was optional and remained anonymous. All preparatory material was shared with all students following the completion of the data collection. Ethical approval was obtained from the Ethics Review Committee of Kotelawala Defence University.

### Material for pre-skill conceptualisation

Preparatory material was posted in the Google classroom 1 week prior to the NR FSL. CK was presented identically for both groups while PK was presented in one of the two forms as shown in Table 1. The students were instructed not to share or discuss the material amongst themselves. All the material was based on the Newborn life support course manual published by the Sri Lanka College of Paediatricians [35]. The 3 DVs were custom-made by the authors, and included a step-by-step demonstration of newborn care and resuscitation with narration and a viewing duration of 10 min for the 3 videos in total. The PP presentation consisted of 82 slides covering the same material as the videos, but in text and images. In both cases, students were able to go through the material at their own pace, rewinding or fast forwarding the video and going back to a slide or skipping a slide if needed. Persky & MacLaughlin’s (2017) synthesis of evidence regarding reading speed and comparison of the time taken to either read or watch/listen to and understand new material, allowed a comparison of the two types of material. At a reading speed of 400 words per minutes it would take approximately 27.3 s to read 82 subject matter slides of approximately 20 words per slide. If it takes 3 times longer to read and understand than it takes to watch/listen and understand, the weight of the PP and the video in terms of study time could be considered equivalent for both groups.

**Table 1:** Comparison of pre-session material for both groups

Preparatory material	Type	Traditional group	Interventional group
List of equipment and consumables required for normal newborn care and resuscitation	Reading material	+	+
Physiology of transition to extrauterine life	Reading material	+	+
Basic newborn care at delivery	Reading material	+	+
Steps of newborn resuscitation	PP with text and images	+	_
	Three custom-made demonstration videos	-	+

PowerPoint(PP)

### Skills session

The 3-station simulated skills session was held at UHKDU in February 2022 (TG) and March 2022 (IG). The students (n = 40) in each cohort were grouped into 3 subgroups of 13 or 14 by an administrative assistant based on the pre-test scores so as to ensure a normal distribution within each group with respect to baseline knowledge in an attempt to ensure team diversity and optimal group dynamics [36]. The NR algorithms and essential drug doses were displayed on posters at each skill station as visual cues. Station specific checklists of the steps of NR were provided to each student (Additional file 1). Each skill station had equipment and low or medium fidelity mannequins for two students to practice in tandem. For the TG, at each station the instructor gave a demonstration during the first 15 min, following which students spent up to 10 min each practicing with peer observation and feedback. No demonstration was given for the IG and students proceeded directly to 12–13 min each of hands-on practice at each station. Other than the instructor demonstration and shorter duration for hands-on practice per student in the TG, the conduct of the skills session was similar in both groups. Rapid Cycle Deliberate Practice, a modification of the deliberate practice model which is less time-consuming because of rapid cycling between practice and feedback within the training event [37] was used for hands-on practice. A single instructor circulated amongst the students within each station for both groups, clearing doubts, correcting mistakes and providing just-in-time information and feedback. Instructors were either specialist consultants or lecturers certified in NR. Additional near-peer support was also offered at each station by 3 senior students who had undergone NR training the year before.

## Data collection

### Pre-interventional measures

All students completed a pre-test consisting of 20 single best answer questions which was shared as a Google form 1 day prior to the skills session. Ten questions each tested CK and PK, with questions arranged in a random order (Additional file 2).

### Post-intervention measures

Following the skills session, all students were assessed on one integrated simulated scenario created by the authors based on authentic clinical situations. These scenarios amalgamated the competencies acquired in each of the three stations. Individual performance was assessed using a checklist created by the authors, content validated by a Neonatologist and a Paediatrician and piloted on 2 previous student groups. (Additional file 3). Skills assessments were done by the same instructors involved in the skills session. Responses to the post-test comprising the same questions as the pretest but with shuffled responses were collected for up to 48 h after the skills session. Students were also invited to fill in the student perception survey. Students rated all aspects of the NR FSL and responded to a free-answer question on their opinions of the FSL and suggestions for improvement.

### Data analysis

Of the 80 students in the student batch, 45 students consented to participation (56%), comprising 22 from the February cohort (TG) and 23 from the March cohort (IG). Four of the data sets were incomplete and were excluded from data analysis. Thus, 41 data sets comprising of data of 19 students from the TG and 22 students from the IG were analysed. SPSS v.25 was used for the analysis of quantitative data. Conceptual and procedural knowledge sub-scores were computed for each student by adding marks of relevant questions, with the sum of both sub-scores representing the total knowledge score. Performance in the simulated integrated scenario was assessed after the FSL and the score marked out of 30 events on the checklist was converted to a percentage. Due to the small sample size, non-parametric tests were used. Mann–Whitney U test was used to compare scores between groups while within groups pre-test and post-test scores were compared using Wilcoxon-Signed Rank test. Given that non-parametric tests were used, the z value of each test was used to calculate the effect size [38] in the form of Cohen’s r [39], in which 0.5 is considered a large effect size and 0.3 a medium one [40]. A p value less than 0.05 was considered statistically significant. Content and thematic analysis was performed on the responses received for the free answer question by two authors (FY and SS) to identify deductive codes, which were then aggregated into manifest themes, following the steps suggested by Joffe and Yardley [41]. The anonymised datasets used for statistical analysis are included as Additional files 4 and 5.

## Results

### Descriptive statistics

The sample demonstrated similar age distribution with a female predominance in both groups (Table 2). This reflects the female predominance (64.1% of all admissions) in the national gender statistics of admissions to government universities and higher education institutions in the year 2019/20 [42].

**Table 2:** Profile of the study sample

Variable	Level	Study group					
		Traditional		Experimental		Total	
		No	%	No	%	No	%
Gender	Female	12	63.2	15	68.2	27	65.9
	Male	7	36.8	7	31.8	14	34.1
Age	22–25 years	4	21.1	5	22.7	9	22.0
	26–29 years	15	78.9	17	77.3	32	78.0
	Total	19	100.0	22	100.0	41	100.0

### Knowledge and skills acquisition

Tables 3 and 4 show the comparisons of collected measures between groups and within groups respectively. Baseline pre-test total knowledge scores between the groups did not differ significantly (Table 3). Between groups analysis of sub-scores showed that the baseline CK of both groups also did not differ significantly. However, the TG scored significantly higher in the PK component of the pre-test than the IG (TG = 7.32, SD = 1.83; IG = 6.09, SD = 1.90; *p* < 0.05). While the difference in post-test total scores between groups did not reach statistical significance, both groups demonstrated a significant gain in total knowledge following the FSL with moderate effect sizes (TG: *p* < 0.05, r = 0.37; IG: *p* < 0.005, r = 0.47) (Table 4). Sub-score analysis showed that while the gain in CK in both groups showed significant increase with moderate effect sizes (TG; *p* < 0.01, r = 0.45, IG; *p* < 0.01, r = 0.40), the gain in PK reached statistical significance only in the IG (*p* < 0.01, r = 0.44)). Finally, skill performance was significantly better (*p* < 0.05) in the IG (M = 87.86%, SD = 5.89) than in the TG (M = 83.44%, SD = 5.30), with a medium effect size (r = 0.40).

**Table 3:** Comparison of test and scenarios scores between groups

Intervention	Test variable	Study group		Test statistics	Significance (p)	Effect size (r)
		Traditional (N = 19)	Experimental (N = 22)			
		Mean (SD)	Mean (SD)			
Pre	Conceptual knowledge	7.84 (1.77)	7.82 (1.92)	U = 207.000 z = -0.053	0.957	0.01
	Procedural knowledge	7.32 (1.83)	6.09 (1.90)	U = 127.500 z = -2.162	0.031*	0.34
	Total knowledge	15.16 (2.95)	13.91 (3.42)	U = 153.500 z = -1.473	0.141	0.23
Post	Conceptual knowledge	9.11 (0.88)	9.36 (1.18)	U = 153.000 z = -1.611	0.107	0.25
	Procedural knowledge	7.68 (1.63)	7.72 (1.28)	U = 208.000 z = -0.027	0.978	0.004
	Total knowledge	16.79 (2.15)	17.09 (2.20)	U = 190.000 z = -0.506	0.613	0.08
	Integrated Scenario	83.44 (5.30)	87.86 (5.89)	U = 112.000 z = -2.544	0.011*	0.40

SD: Standard Deviation, *p < 0.05

**Table 4:** Comparison of test scores within groups

Student group	Test variable	Intervention		Test statistics (z)	Significance (p)	Effect size (r)
		Pre	Post			
		Median (IQR)	Median (IQR)			
Traditional (N = 19)	Conceptual knowledge	8.0 (7–9)	9.0 (9–10)	-2.770	0.006**	0.45
	Procedural knowledge	7.0 (7–9)	7.0 (7–9)	-0.926	0.355	0.15
	Total knowledge	16.0 (13–17)	17.0 (15–19)	-2.289	0.022*	0.37
Experimental (N = 22)	Conceptual knowledge	9.0 (6–9)	10.0 (9–10)	-2.677	0.007**	0.40
	Procedural knowledge	6.0 (4.75–7)	8.0 (7–9)	-2.915	0.004**	0.44
	Total knowledge	14.5 (11–16)	17.0 (17–19)	-3.141	0.002**	0.47

IQR: Interquartile Range, *p < 0.05, **p < 0.01

### Student views and perceptions

Twenty nine students responded to the student survey, 16 from the TG and 13 from the IG. All students had a positive impression of the NR program with an overall rating of 4.46 in the IG and 4.54 in the TG (rated on a scale of 1- 5, where 1 represented very poor and 5 represented excellent). More than 90% of students in both the groups either strongly agreed or agreed that the preparatory material was interesting and engaging (TG: strongly agreed = 68.8%, agreed = 25.0%; IG: strongly agreed = 61.5%, agreed = 30.8%). More students in the IG (84.6%) strongly agreed that the pre-session material was easy to understand than students in the TG (62.5%). All the students in the IG self-reported going through all the material in full, while only 75% of the students in the TG reported completely reviewing the preparatory material.

Following the FSL, more students strongly agreed that the DVs were useful for hands-on practice (84.6%) than the PPs (68.8%). A fair gain in knowledge was self-perceived in more than two times the number of students in the IG than the TG (TG = 6.3%, IG = 15.4%). However, around 10% more students in the TG reported superior gains (improved a lot/improved quite a lot) in self-perceived knowledge than in the IG (TG = 93.8%, IG = 84.7%). While the number of students who reported feeling fairly confident in handling a newborn delivery following the FSL was similar in both the groups (TG = 62.5%, IG = 61.5%), the number students in the IG who reported feeling extremely confident in handling a newborn delivery was almost twice the number in the TG (TG: 18.8%, IG = 30.8%).

Content and thematic analysis of free responses revealed 3 major themes, value for learning, interactive nature and learning experience. Given the straightforward nature of the single question and small volume of data, the codes identified by both authors were similar. Students valued the hands-on and interactive nature of the NR FSL, but felt it was too long. Students described the session as a great experience in learning and suggested that the FC approach should be applied to other areas as well.

## Discussion

Comparable baseline levels of CK in both groups can be expected as pre-skill conceptualisation of CK was identical. However, the results failed to show that DVs alone are superior to PPs in acquisition of PK as demonstrated by the relatively lower pre-test PK sub-score in the IG (despite students reporting higher engagement and ease of understanding) than that of the TG. This can be explained by the relative unfamiliarity with independent learning from DVs as opposed to the more commonly used PP. This explanation may also reflect the lower level of perceived knowledge gain in the IG when compared with the TG. Despite this, the overall learning gain was similar in both groups following the FSL. The gain in CK following the FSL in both groups suggests that CK is well received and gained using text-based material and supplemented by hands-on training. However, despite being less knowledgeable in PK than the TG before the skills session, PK-specific knowledge gains, as well as skill performance were superior in the IG following the FSL. This confirms that using short, segmented DVs to share PK is more effective than text and image-based material in a flipped approach to teaching complex skills like NR. Taken together, the results suggest that the combination of text-based CK and video-based PK as pre-skill conceptualisation complement hands-on practice and results in superior NR skills acquisition than text-based CK and text and image-based PK with hands-on practice.

The use of DVs in a FSL can mediate this benefit in several ways, the most obvious being that offloading demonstration to before the skill session, obliviates the need for in-class demonstration. While there is some evidence that learning gains in the FC are better if in-class time is not curtailed [4], any time gained due to asynchronous preparation and demonstration in the context of PS training will be useful for students by providing more time for repeated hands-on practice using frameworks such as deliberate practice, which in itself can improve outcomes [15]. However, further analysis of the results of our study shows that the benefits may extend beyond temporal reasons.

Pedagogically informed instructional DVs can provide cognitive and emotional benefits for learning [43]. Firstly, based on the principle of modality of the Cognitive theory of Multimedia Learning [44], the spoken word (narration) is more educationally efficient than the printed word. Secondly, the principle of multimedia explains how the simultaneous processing of the complementary audio and video signals which result in the creation of inter-connected verbal and visual mental models, enhances the germane load of the learning experience. Thirdly, repeated, self-paced viewing and iterative review and modification of this mental model occurs during the period of preparation before hands-on practice. This is important not only because pre-created mental models are useful for learning [29], but also because regular and steady engagement with preparatory material has been found to be useful in the FC [45]. Finally, the principle of segmentation of the SSW model for the design of instructional videos supports the use of segmented DVs which permits division of the intrinsic load of a task, thus enhancing learning gains [46].

The preference of medical students for digital resources [47] explains why all students in the IG reported going through the preparatory material in full. Further, medical students have been shown to view DVs positively, finding such videos effective for learning PS [48]. Despite objective measures of PK gain falling short in the IG just after pre-skill conceptualisation, students reported acceptable self-perceived gains in knowledge following the skills lab. The superior objective gains in PK as well as skill performance in the IG seen after hands-on practice during the skills session support the proposal that preparatory material that is tailored and complementary to the learning objectives of the session positively influences outcomes [21].

Finally, according to Illeris’ holistic theory of learning, there is a bi-directional influence of motivation, emotion and volition (incentive) on learning [49]. In other words, students learn better if they feel engaged and motivated. Reciprocally, feeling motivated and engaged can positively influence learning. Similar effects are attributed to a feeling of confidence [50]. Thus, the superior skill performance in the IG may be explained by the additional influence of pedagogically informed preparation on performance through its positive influence on motivation, engagement and self-perceived confidence in the EG.

This study was limited to one centre with a small sample and thus results may not be generalizable. Since the sample was one of convenience, selection bias may exist in that only those students who were interested and motivated consented to the study. Thus, larger, well controlled studies are required to confirm the results. Other directions to be studied include specific characteristics of the flipped approach that could influence learning such as duration required for preparation and volume of preparatory material. While this study measured only short-term knowledge and skills acquisition, future studies should look into how the design of the FSL can influence long-term retention and transfer of skills to practice.

## Conclusions

This study shows how a flipped approach in the form of a FSL for NR training was well received by medical students and led to considerable knowledge and skills acquisition. More importantly, the results show how a combination of pedagogically informed material for pre-skill conceptualisation including PK in the form of short, segmented DVs with step-by-step narration complements hands-on practice. Relevant educational theory shows how such considerations can boost outcomes on temporal, cognitive and emotional bases.

## Supplementary Information

**Additional file 1: Supplementary file 1.**

**Additional file 2: Supplementary file 2.**

**Additional file 3: Supplementary file 3.**

**Additional file 4: Supplementary file 4.**

**Additional file 5: Supplementary file 5.**

## Data Availability

The materials described in the manuscript are included as Additional files 1–3. The data sets generated during and analysed during this study are included as Additional files 4 and 5.

## Abbreviations

CK: Conceptual knowledge
DVs: Demonstration videos
FC: Flipped classroom
FSL: Flipped skills lab
IG: Interventional group
NR: Newborn resuscitation
PPs: PowerPoint presentations
PK: Procedural knowledge
PS: Procedural skills
TG: Traditional group
