# Correction: Videos of demonstration versus text and image-based material for pre-skill conceptualisation in flipped newborn resuscitation training for medical students: a pilot study

**DOI:** 10.1186/s12909-022-03963-x

**Published:** 2022-12-20

**Authors:** Farah Yoosoof, Indika Liyanage, Ranjith de Silva, Savindra Samaraweera

**Affiliations:** 1 Department of Paediatrics, General Sir John Kotelawala Defence University, Rathmalana, 10390 Sri Lanka; 2 Sri Lanka College of Obstetricians and Gynaecologists, No.112 Model Farm Rd, Colombo, Sri Lanka; 3 Leeds General Infirmary, Leeds, UK

**Correction: BMC Medical Education (2022) 22:839**

**https://doi.org/10.1186/s12909-022-03926-2**

Following publication of the original article [1], the authors identified the following errors in the Abstract

(1) In the Methods section the line ‘Students in the traditional group (n = 19) and those in the interventionalmental group (n = 22) received identical reading material’, the incorrectly typed word ‘interventionalmental’ should be replaced with the word ‘interventional’. The full corrected sentence should read: ‘Students in the traditional group (n = 19) and those in the interventional group (n = 22) received identical reading material covering conceptual knowledge’.

(2) In the Results section, the sentence ‘Post-intervention skill performance in the experimental group was significantly better (*p* < .05) in the interventional group (M = 87.86%, SD = 5.89)’, should read as: ‘Post-intervention skill performance was significantly better (*p* < .05) in the interventional group (M = 87.86%, SD = 5.89) than in the traditional group (M = 83.44, SD = 5.30) with a medium effect size (r = .40)’. Where the word ‘experimental group’ was mentioned in the two instances following this sentence, it should be replaced with the words ‘interventional group’.

The authors would like to apologise for these errors which had been inadvertently overlooked during the final proof reading.
