# Supplementary material for: Videos of demonstration versus text and image-based material for pre-skill conceptualisation in flipped newborn resuscitation training for medical students: a pilot study
Source: BMC Med Educ. 2022 Dec 5;22:839. doi: 10.1186/s12909-022-03926-2 (PMC9721000; doi:10.1186/s12909-022-03926-2)
Supplement: Supplementary file 1 — Additional file 1: Supplementary file 1. [file 12909_2022_3926_MOESM1_ESM.pdf]

Additional file 1. Station specific checklists

STATION 1 - GETTING READY FOR A DELIVERY/EQUIPMENT CHECK/ROUTINE NEWBORN CARE

	YOU HAVE BEEN CALLED TO LR FOR A VACUUM DELIERY OF A TERM BABY				
		1ST ATTEMPT		2ND ATTEMPT	
	PERFORMANCE	YES	NO	YES	NO
1	Closes windows				
2	Switches off AC/fan				
3	Switches on warmer				
4	Checks Oxygen supply				
5	Checks suction				
6	Checks for consumables (OP airway/suction tube/syringes/UVC/NG tube/ET tubes/Vit K /other drugs)				
7	Prepares and labels Adrenalin *				
8	Sanitises/Washes hands using 5 steps				
9	Double gloves				
10	Towels for warming				
12	Checks Ambu bag (pressure valve/pop off valve)				
13	Checks masks				
14	Checks laryngoscope				
15	Removes outer gloves when baby is born				
16	Starts the clock				
17	Receives with dry prewarmed towel				
18	Places on firm flat surface				
19	Dries head, then limbs and body				
20	Discards wet towel				
21	Wraps in 2nd dry prewarmed towel /covers head/places cap				
22	Keeps chest exposed				
23	Gives instructions to connect to pulse oximeter				
24	Maintains neutral position				
25	Assesses using CTBH				
COLOUR – PINK BODY , BLUE PERIPHERIES/ TONE- FLEXED EXTREMITIES/ BREATHING – YES/ HEARTRATE > 100 BPM					
26	Applies cord clamp				
27	Administers correct dose of Vit K				
28	Recognises need to maintain normothermia				
COLOUR – PINK/ TONE- FLEXED EXTREMITIES/ BREATHING – YES/ HEARTRATE > 100 BPM					
29	Wraps and hands over to mother				
30	Calculates and documents APGAR				

STATION 2 - AIRWAY OPENING MANOUVERS/SUCTION/INFLATION BREATHS

You have just arrived at the LR after being called because a baby has not cried after delivery. The baby is on the warmer , already wrapped in a dry prewarmed cloth with the head covered and chest exposed					
	PERFORMANCE	1ST ATTEMPT		2ND ATTEMPT	
		YES	NO	YES	NO
1	Maintains in neutral position				
2	Assesses CTBH				
COLOUR– BLUE/ TONE - FLOPPY / BREATHING – NOT BREATHING/ HR <100 BPM					
3	Applies jaw thrust (double handed)				
4	Assess CTBH				
COLOUR– BLUE/ TONE - FLOPPY / BREATHING – NOT BREATHING/ HR <100 BPM					
5	Selects correct size OP airway (*)				
6	Inserts OP airway correctly (*)				
7	Assess CTBH (*) *May skip				
8	Chooses correct size mask				
9	Correctly fits mask onto the AMBU bag				
10	Demonstrates C and E technique with single handed jaw thrust<br>OR<br>Demonstrates C +/- E technique without/with OP airway<br>OR<br>Calls for help and holds mask in place with a good seal with double C and double handed jaw thrust OR with OP airway				
11	Delivers Inflation breaths * 5 using correct technique/count				
12	Reassess CTBH				
COLOUR– BLUE/ TONE - FLOPPY / BREATHING – NOT BREATHING/ HR <100 BPM					
13	Confirms chest expansion				
CHEST EXPANSION NOT ACHIEVED/ GURCLING SOUNDS HEARD					
14	Selects correct laryngoscope				
15	Selects correct sized suction tube				
16	Sets suction pressure at 80 - 100				
17	Inserts laryngoscope with left hand correctly				
OFFER THAT THERE ARE VISBLE SECRETIONS					
17	Sucks out secretions under direct vision				
18	Assesses CTBH				
COLOUR– BLUE/ TONE - FLOPPY / BREATHING – NOT BREATHING/ HR <100 BPM					
19	Delivers Inflation breaths * 5 using correct technique/count				
20	Assesses CTBH				
21	**AT ANY ONE TIME ASSESSES MR-SOPA**				
COLOUR– IMPROVED/ TONE - SOME TONE +/ BREATHING – NOT BREATHING/ HR >100 BPM					
	Confirms chest expansion				
CHEST EXPANSION ACHIEVED/ BABY NOT BREATHING					
21	Chooses correct size ET tube				
22	Chooses correct laryngoscope				
23	Inserts ET tube				
24	Conforms position of ET tube				
25	Secures ET tube				

STATION 3 : COORDINATION OF VENTILATION BREATHS WITH CHEST COMPRESSION/CIRCULATION/DRUGS

NAME : ____

You have just arrived at the OT after being called because a baby has not cried after delivery.<br>A nurse is maintaining the baby in the neutral position and has just given 5 inflation breaths with good chest movement. She says that the baby's colour has improved after the inflation breaths and she has achieved chest expansion<br>On assessment you see – COLOUR – SOME PERIPHERAL CYANOSIS/ TONE – FLOPPY/ BREATHING – NOT BREATHING/ HEART RATE – SLOW < 60 BPM					
		1ST ATTEMPT		2ND ATTEMPT	
	PERFORMANCE	YES	NO	YES	NO
1	Recognises need to move to ventilation breaths				
2	Recognises need to initiate chest compressions				
3	Instructs assistant to proceed to ventilation breaths				
4	Identifies correct position for chest compressions				
5	Performs chest compressions using chest encircling technique with proper depth of compression				
6	Performs chest compression: ventilation at 3: 1 for 30 breaths (60 s)				
7	Reassess CTBH after 30 breaths				
COLOUR– SOME CYANOSIS/ TONE - FLOPPY / BREATHING – NOT BREATHING/ HR <60 BPM					
CHEST EXPANSION PRESENT					
8	Continues with CC:VB at 3:1 for 30 seconds (15 breaths)				
9	Assess CTBH				
COLOUR– SOME CYANOSIS/ TONE - FLOPPY / BREATHING – NOT BREATHING/ HR <100 BPM					
10	Continues with VB:CC at 3:1 for 30 seconds				
11	Assess CTBH				
COLOUR– SOME CYANOSIS/ TONE - FLOPPY / BREATHING – NOT BREATHING/ HR <60 BPM					
12	Recognises the need for vascular access				
13	Calls for senior help				
14	Instructs to continues with CC:VB ratio 15:1				
15	Prepares equipment for UVC insertion				
16	Prepares Adrenaline 1:10000				
17	Places cord tie at base of stump				
18	Primes UVC/NG tube with normal saline and connected syringe				
19	Cuts stump at least 3 cm above base				
20	Identifies vessels correctly				
21	Inserts UVC till black flow seen (3-5 cm)				
22	Administers Adrenaline 1:10000 0.1 -0.3 ml/kg via UVC				
23	Flushes with N/S				
24	Reassess CTBH				
COLOUR– BLUE/ TONE - FLOPPY / BREATHING – NOT BREATHING/ HR <100 BPM/ CRFT>4 S					
25	Confirms chest expansion				
26	Continues with VB:CC at 3:1 for 30 seconds				
27	Administers N/S bolus 10 ml/kg via UVC				
28	Coordinates continuation of good quality resuscitation				
29	Calls for senior help				
