# Supplementary material for: Videos of demonstration versus text and image-based material for pre-skill conceptualisation in flipped newborn resuscitation training for medical students: a pilot study
Source: BMC Med Educ. 2022 Dec 5;22:839. doi: 10.1186/s12909-022-03926-2 (PMC9721000; doi:10.1186/s12909-022-03926-2)
Supplement: Supplementary file 2 — Additional file 2: Supplementary file 2. [file 12909_2022_3926_MOESM2_ESM.pdf]

Additional file 2. Sample single best answer questions testing conceptual and procedural knowledge

| EXAMPLES OF PRE-TEST AND POST-TEST SINGLE BEST ANSWER QUESTIONS | |
|---|---|
| TESTING CONCEPTUAL KNOWLEDGE | TESTING PROCEDURAL KNOWLEDGE |
| 1] What DIFFERENTIATES terminal apnea from primary apnea?<br>A) Terminal apnea means that the baby is not breathing<br>B) In Terminal apnea the heart rate starts to drop<br>C) Terminal apnea blood pressure drops drastically<br>D) Terminal apnea usually responds to stimulation promptly | 1] A term baby is born who is blue, floppy, not breathing, and with a heart rate <100 bpm. You dry while stimulating, and place the head in the neutral position. After applying double sided jaw thrust and securing the airway, you find that he has not improved and is not breathing. What is the immediate next step that you should take to assist the baby to transition to extrauterine life?<br>A) Call for help. This baby needs immediate Endotracheal intubation<br>B) Stimulate the baby again by rubbing vigorously on the back and feet<br>C) Deliver 5 inflation breaths via 450 ml AMBU bag with air<br>D) Deliver 5 inflation breaths via 250 ml AMBU bag with Oxygen |

| | |
|---|---|
| 2] Inflation breaths:<br>A) Cannot be delivered effectively without a proper Oxygen supply<br>B) Push the alveoloar fluid out gradually and ensure lung expansion<br>C) Can only be performed by highly skilled doctors to be administered<br>D) Should be counted off as 1-and 2-and 3 and 4 and 5 and... | 2] A term newborn has been resuscitated with chest compressions and ventilation breaths via ET tube at the rate of 3: 1 for 60 seconds. You auscultate the heart rate which is 40 bpm and baby appears dusky. What is the next step that should be considered in this baby?<br>A) Check if ET tube in situ by Chest X Ray<br>B) Discontinue chest compressions and further resuscitation<br>C) Give 0.2 ml/kg of Adrenaline 1:1000 via ET tube<br>D) Get ready to obtain venous access via the umbilical vein |
