# Supplementary material for: Videos of demonstration versus text and image-based material for pre-skill conceptualisation in flipped newborn resuscitation training for medical students: a pilot study
Source: BMC Med Educ. 2022 Dec 5;22:839. doi: 10.1186/s12909-022-03926-2 (PMC9721000; doi:10.1186/s12909-022-03926-2)
Supplement: Supplementary file 3 — Additional file 3: Supplementary file 3. [file 12909_2022_3926_MOESM3_ESM.pdf]

Additional file 3. Sample Integrated Simulated Scenario

You have been called to the LR to see a term baby who has not cried after birth. The N/O has dried the baby and wrapped in a dry cloth with head covered and chest exposed.					
	PERFORMANCE	YES	NO	WITH PROMPT	NOT EVEN WITH PROMPT
1	Wash hands/Sanitize				
2	Wear sterile gloves				
3	Maintain neutral position				
4	Connect to Pulse oximeter *				
5	Assesses CTBH				
COLOUR– BLUE/ TONE - FLOPPY / BREATHING – NOT BREATHING/ HR <100 BPM					
6	Applies double handed jaw thrust				
7	Assess CTBH				
COLOUR– BLUE/ TONE - FLOPPY / BREATHING – NOT BREATHING/ HR <100 BPM					
8	Selects correct size OP airway (*)				
9	Inserts OP airway correctly (*)				
10	Assess CTBH (*) *May skip				
COLOUR– BLUE/ TONE - FLOPPY / BREATHING – NOT BREATHING/ HR <100 BPM					
11	Chooses correct size mask				
12	Correctly fits mask onto the AMBU bag				
13	Demonstrates C and E technique with single handed jaw thrust<br>OR<br>Demonstrates C +/- E technique with/without OP airway<br>OR<br>Calls for help and holds mask in place with a good seal and with double handed jaw thrust or with OP airway				
COLOUR– IMPROVED MARGINALLY - FLOPPY/ BREATHING – NOT BREATHING / HR <60 BPM					
14	Checks for chest expansion				
CHEST EXPANSION ACHIEVED					
15	Calls for help				
16	Delegates ventilation breaths to assistant				
17	Identifies correct position for chest compressions				
18	Verbalizes depth of compressions				
19	Delivers chest compressions using the correct technique				
20	Delivers CC: VB ratio of 3:1 for 30 breaths (60 sec)				
21	Reassess CTBH				
COLOUR– SAME AS BEFORE / TONE - FLOPPY/ BREATHING –NOT BREATHING / HR<60 BPM					
22	Recognises the need for intubation and continuation of CC:VB				
23	Calls for help				
24	Chooses correct size ET tube				
25	Chooses and assembles correct laryngoscope				
26	Inserts laryngoscope				
27	Inserts ET tube to correct position				
28	Confirms position				
29	Secures ET tube				
30	Continues good quality CC:VB				
